# The Italian COVID-19 Psychological Research Consortium (IT C19PRC): General Overview and Replication of the UK Study

**DOI:** 10.3390/jcm10010052

**Published:** 2020-12-25

**Authors:** Giovanni Bruno, Anna Panzeri, Umberto Granziol, Fabio Alivernini, Andrea Chirico, Federica Galli, Fabio Lucidi, Andrea Spoto, Giulio Vidotto, Marco Bertamini

**Affiliations:** 1 Department of General Psychology, University of Padua, 35122 Padova, Italy; giovanni.bruno.4@phd.unipd.it (G.B.); anna.panzeri@phd.unipd.it (A.P.); umberto.granziol@unipd.it (U.G.); andrea.spoto@unipd.it (A.S.); giulio.vidotto@unipd.it (G.V.); 2 Department of Developmental and Social Psychology, University of Rome, 00185 Roma, Italy; fabio.alivernini@gmail.com (F.A.); andrea.chirico@uniroma1.it (A.C.); federica.galli@uniroma1.it (F.G.); fabio.lucidi@uniroma1.it (F.L.); 3 Department of Psychological Sciences, University of Liverpool, Liverpool L69 3BX, UK

**Keywords:** COVID-19, psychological health, general population, somatic symptoms, anxiety, depression

## Abstract

The COVID-19 pandemic represents a major stressor for the psychological health of people worldwide. In the UK, the COVID19-Psychological Research Consortium Study (C19PRC) launched to evaluate the psychological impact of COVID-19 in the general population and its implications. The project was then extended to Italy and several other countries. This article provides an overview of the Italian C19PRC study and its replication of two specific findings from the UK C19PRC. In the first part, the relationship between anxiety and somatic symptomatology is examined. In the second part, we analyze the association between several factors and psychological health outcomes: depression/anxiety, traumatic stress, COVID-19 anxiety. In line with the study conducted in the UK, an online survey was administered to the adult Italian general population. The sample included 1038 respondents (age, mean = 49.94, SD = 16.14, 51.15% females) taken from four regions: Lombardia, Veneto, Lazio, and Campania. The relationship between predictors and outcomes was evaluated by means of logistic regression models. Somatic indices showed a positive association with anxiety, worse somatic symptoms were associated with mourning a loss of a beloved one due to COVID-19 and with precarious health conditions. Females showed a higher incidence of psychological issues. No differences in anxiety, depression, and traumatic stress were found across regions but the Campania region showed the most severe somatic symptomatology. In the second analysis, the factors associated with more severe psychological outcomes (i.e., anxiety and/or depression, traumatic stress, and COVID-19 related anxiety) were younger age, the presence of minors in the household, traumatic stressors, and precarious health conditions. No differences across regions emerged. The Italian results correspond to the UK findings for anxiety, depression, and traumatic stress. Both in the UK and Italy, the factors associated with worse psychological health were gender (female), younger age, having children, pre-existing health issues (both for oneself or someone close), and the moderate/high perceived risk of contracting COVID-19 within one month. In Italy, unlike the UK, lower household income and having (had) COVID-19 were not associated with poorer mental health. The psychological impact of COVID-19 can last for months; future research should explore all aspects of the psychological burden of COVID-19 in order to implement psychological interventions and promote psychological health.

## 1. Introduction

Coronavirus-19 (COVID-19) is a virus first identified in 2019 that has led to an ongoing health emergency worldwide, with high infection rates and mortality [1]. The World Health Organization recognized the outbreak as a Public Health Emergency of International Concern in January 2020, and as a pandemic in March 2020. Infection can cause systemic organ disease and some categories of people are more at risk in developing severe health consequences, such as those over 70 years old, pregnant women, and those who have pre-existing health conditions (e.g., cardiovascular disease) [2]. However, everyone can be infected and the long-term health consequences—also in its milder presentations—are still not fully understood.

Given the high transmissibility of COVID-19, to contain contagion many countries have adopted restrictive measures such as lockdown, quarantine, social distancing, and limits to movements and travel. These measures have serious economic as well as social implications. Therefore, these aspects of the pandemic extend beyond the health domain. The lives of millions of people have been affected, potentially increasing anxiety, loneliness, and distress. Some of these behavioral responses have been noted and commented in the media. Moreover, the measures have produced various responses, ranging from resistance, stockpiling of food supplies, denial, and beliefs in conspiracy theories. Some behavioral changes have the potential to affect the course of the pandemic itself [3].

The impact of the pandemic can be analyzed from social, psychological, and economic perspectives. Some authors have claimed that we are in the presence of a mental health emergency [4]. Over time fear, anxiety, worry, and depression have grown among people [5]. According to some, the psychological impact of COVID-19 may be greater than the threat represented by the physical disease itself, especially for vulnerable individuals [6]. A growing number of studies have highlighted psychological symptoms and issues in the clinical and general population [7,8,9]

A focus on the clinical population and on specific risk categories (e.g., young adults, pregnant women, health professionals, caregivers, the elderly) is useful to highlight the needs of these groups [10,11,12]. However, at the same time, it is important to direct attention to the general population to identify psychological and behavioral patterns.

Several studies have been conducted during the acute phase when contagions reached their peak. The effect of stressors and the psychological symptoms may persist or evolve over periods or months [13]. This is the case when people experience fear (for self and for significant others), stigmatization, and severe psychological symptoms [14].

Italy was one of the first countries to face the COVID-19 emergency and in an especially intense manner. In China, the city of Wuhan was put under quarantine on the 9th of January 2020. Already on/by the 21st of February 16 cases were confirmed in Lombardia and two in Veneto (two Italian regions in the North). Cases grew quickly leading to shortfalls of hospital beds, especially in intensive care units (ICU). The impact on the individual, social, and economic life in Italy was significant. In Italy, as in other countries, the pandemic did not affect all regions equally. Thus, national statistics can be misleading. An analysis by region is important for at least two reasons: first, the north of Italy is divided in eight regions but there are large differences in the impact even between these. Second, in Italy the national health service has a regional structure, and different measures were taken by different regions, (e.g., different testing effort, quarantine policy, individual mobilities, the progression of social distancing, and local capacity of medical infrastructure) [15]. Third, the timeline of the virus varied by region. Lombardia was the first region to be affected and remained also the region with most cases over time. This is a wealthy region with high population density and its capital Milan is the second largest city in Italy.

In March 2020, a longitudinal, multi-country project was launched by the COVID-19 Psychological Research Consortium (C19PRC) in the UK and then enlarged to other countries [16]. The present study represents the opening article of the Italian C19PRC project. Data was collected in Italy from 13 to 28 July 2020, after the contagion peak (end of March) and after the end of the strict national lockdown (18 May). Many commercial and social activities had restarted, and people were allowed to move beyond their own towns. In mid-July, the number of contagions had a stable and decreasing trend, but there were still preventive measures in place (e.g., social distancing, hygiene practices, masks). There was an awareness in the population that a second wave of contagions was possible in all regions.

This study focused on four Italian regions selected because of their geographical location, from the north (Lombardia, Veneto), center (Lazio), and south (Campania), and because of their infection rate when the survey was launched—at the beginning of July 2020—from higher (Lombardia = 95,118 cases, Veneto = 19,432) to medium-lower (Lazio = 8389, Campania = 4788) [17]. Indeed, when data was collected, from 13 to 18 July, in Italy a total of 243,230 cases of COVID-19 had been registered, with 35,042 deaths. The regions that registered the highest number of contagions were Lombardia and Veneto, while in the center and south the outbreak was more contained.

According to the Italian Ministry of Health, the health services and policies are the same in the Italian regions, but some independent regional choices are allowed. In the North, Lombardia ran fewer tests than Veneto and Emilia Romagna. These regions adopted proactive testing modalities and treatment strategies, testing a large number of people and treating positive patients with mild symptoms at home. Therefore, we chose to compare Lombardia and Veneto. Veneto is geographically next to Lombardia, they both have a mix of large cities and small towns and, with respect to population, Lombardia is the largest (10 million) and Veneto is the 5th largest region in Italy (nearly 5 million). The other two regions also include some large cities (Rome in Lazio and Naples in Campania) and are relatively large (5.8 million each). From 17 May 2020 the government passed responsibility to single regions under the overall supervision of the Ministry of Health [18]; different measures in different regions were motivated by the underlying infections and death trends.

Data from a substantial sample of the general population are useful to plan effective interventions. A range of variables have to be assessed due to the complexity of the psychological, social, and cultural context of the pandemic. Some of them are: demographics; socioeconomic status; political opinions; belongingness in the community; public health knowledge; news broadcasting; risk perceptions; hygienic and preventive practices; decision making (e.g., whether to vaccinate or not). Among the psychological ones, post-traumatic stress, loneliness, anxiety, and depression should be assessed as well as self-esteem, personality, and resilience.

### Aims

This Italian C19PRC study uses data collected on a range of demographic, social, and psychological variables from a sample of the general population in Italy. The aim is to describe the effects of the pandemic in adults. Here we provide an overview of the project, and an initial analysis, while additional detailed analyses will require further work. In particular in this paper we focus on anxiety, depression, and stress related to COVID-19 and on the comparison with the same measurements from the first wave of the UK C19PRC study [19,20]. Moreover, comparisons across four Italian regions—selected according to geographic location and infection rate—are provided.

## 2. Methodological Section

### 2.1. Study Plan

The original complete survey from the UK is described by McBride and colleagues [16]. Our goal was to maintain the Consortium aims and measures, applying them to the Italian population. Part of this data will be used to conduct further in-depth studies focusing on specific topics described in detail in following papers. In line with the UK survey, the Italian C19PRC study relied on an online survey provided by Qualtrics. R software was used for all analyses [21]. The survey was administered in four Italian regions—Campania, Lazio, Lombardia and Veneto. Ethical approval for this study was provided by the Ethical Committee for Psychological Research of the University of Padua (protocol: 3818).

### 2.2. Participants

The inclusion criteria were living in Italy and at least 18 years old. Participants were excluded if survey completion time was below 11 minutes and 11 seconds or above three days. All participants were informed about the study’s aims, their rights, and privacy policies. All of them provided informed consent before completing the survey of the study, conducted according to Ethical Principles and Code of Conduct of the Italian Association of Psychology.

Adult participants *(n* = 1038) were recruited by the survey company Qualtrics from an online research panel using stratified quota sampling to guarantee that the sample characteristics of gender, age, household income, and region (Campania, Lazio, Lombardia, and Veneto) matched the Italian population. After the completion of the survey online (median time of completion = 41 min), each participant was reimbursed by Qualtrics.

The mean age of the total sample was 49.94 years (median = 51, SD = 16.14, range = 18–87), and 51.15% were female (*n* = 531). Participants were recruited from the four selected regions based on their population size: Campania (*n* = 227), Lazio (*n* = 234), Lombardia (*n* = 391), Veneto (*n* = 186). Most of the participants were Italian (96.61%, *n* = 1003) and with Caucasian ethnicity (74.66%, *n* = 775). A minority had only elementary or some secondary education (8.28%, *n* = 86), nearly half had completed high school (48.74%, *n* = 506) with a further 42.97% having attained a higher level of education. Less than half were in full employment (44.41%, *n* = 461), with 24.18% retired (*n* = 251). Married participants comprised 57.99% (*n* = 602) whereas never married participants comprised 26.59% of the sample (*n* = 276).

Only 14 participants tested positive for COVID-19 (1.35%). Two and a half percent of the sample confirmed the presence of coronavirus cases among individuals living in the same house, and 17.82% among friends or relatives. Finally, 10.50% of the participants mourning a loss due to confirmed cases of COVID-19. Further information about demographics is available in the Supplementary Materials (S1, Tables S1–S20 and https://osf.io/nx2zd/).

### 2.3. Measures

In line with the aim of the original C19PRC-UK study, the measures remained the same in order to allow cross-cultural comparison. Below, the measures that are relevant for the present analysis are described. Items were translated into Italian. The full list is available as Supplementary Material (Supplementary File S1 and https://osf.io/nx2zd/).

*Sociodemographic variables*: we collected the same information as the original C19PRC-UK study [16]: location, presence of adults or minors in the house, income, and previous health issues. We integrated these with further information (region, mourning for COVID-19 human losses, perceived risk to contract COVID-19).

*The Patient Health Questionnaire-15* (PHQ-15) [22] is a 15-item self-report measure based on a 3-point Likert scale. It asks about the presence of somatic symptoms in the last 14 days. In this study, we use both the total scores and the four subscales pain symptoms (0–6), gastrointestinal symptoms (0–8), cardiopulmonary symptoms (0–8), and fatigue symptoms (0–4). As in previous works [16,17,18,19,20,21,22,23] and for the same reasons, item 4 and item 13 were excluded, lowering the maximum score to 26.

*The Patient Health Questionnaire-9* (PHQ-9) [24] was used to measure nine symptoms of depression, asking participants how often they have been bothered by each symptom over the last 14 rated on a four-point Likert scale. The maximum score is 27, which is indicative of a high level of depression. A cut-off score of 10 was used, corresponding to moderate levels of depression [25].

*The Generalized Anxiety Disorder 7-item Scale* (GAD-7) [26] is a seven-item self-report measure based on a four-point Likert scale. It asks participants about anxiety symptoms in the last 14 days. The maximum score is 21, which is indicative of high levels of generalized anxiety. A cut-off of 10 was used, corresponding to a moderately severe level of anxiety [27].

*The International Trauma Questionnaire* (ITQ) [28] is a nine-item self-report measure based on a 5-point Likert scale. Consistently with ICD-11 [29], it asks participants about particular events or behaviors in the last 30 days, across the three symptom clusters of re-experiencing, avoidance, and sense of threat (two items each). Another three items measure functional impairment caused by these symptoms. The maximum score is 24, and a score of ≥2 (*Moderately*) is considered endorsement of that symptom. A PTSD diagnosis requires the endorsement of the three clusters and at least one indicator of functional impairment [30,31].

*COVID-19-related anxiety* was measured through a single item, ‘How anxious are you about the coronavirus COVID-19 pandemic?’, rated on a continuous scale from 0 to 100. Higher values correspond to higher COVID-19 related anxiety.

### 2.4. Analytic Methods

In Section 3.1, scores of the COVID-19 anxiety variable were categorized into quintiles, and the quintiles were dummy-coded with the lowest one used as the reference category [20] Regression models were used to estimate the relationship between the COVID-19 anxiety quintiles and the PHQ-15 subscales (pain, gastrointestinal, cardiopulmonary, and fatigue). Model 1 included the four dummy-coded COVID-19 anxiety variables as predictors of the four PHQ-15 subscales. The regression coefficient for each dummy-coded variable is interpreted as the mean difference between each quintile and the lowest one. Model 1 was also run separately with the total PHQ-15 summed scale score replacing the subscales. In Model 2 the covariates (age, gender, income, pre-existing health problems, and GAD) were included as predictors, with the addition of Italian region mourning for COVID-19 losses factors.

In Section 3.2, measures of depression (PHQ-9), generalized anxiety (GAD-7), trauma symptoms relating to the pandemic (ITQ) and COVID-19 anxiety were considered as dependent variables for three binary logistic regression models [19]. The predictor variables were age, gender, living location, living alone, presence of children in the household, income, pre-existing health conditions in self and someone close, and perceived risk of infection over the next month. Italian region and mourning for COVID-19 losses factors were included.

## 3. Results

### 3.1. Somatic Symptoms

**Figure 1:**
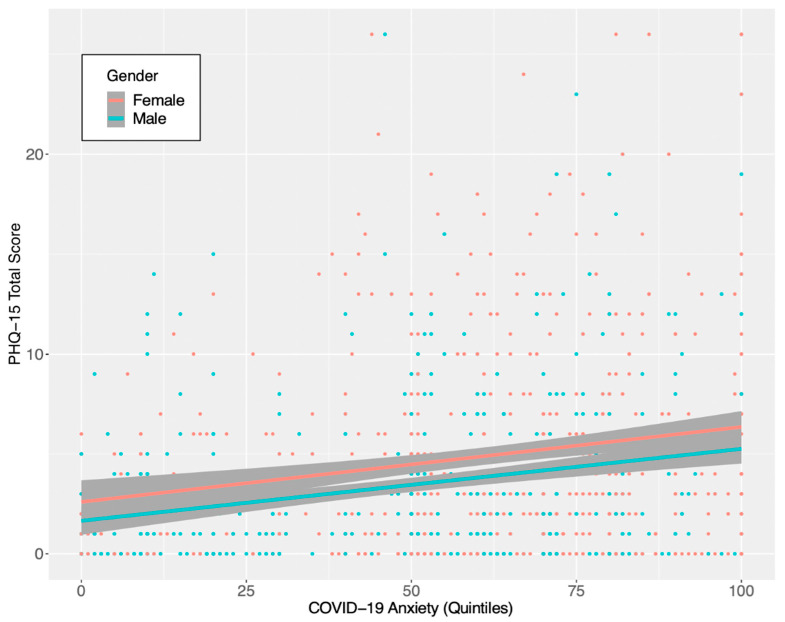
Relationship of COVID-19 anxiety on somatic symptoms (Model 2).

Correlations for all the variables included in Models 1 and 2 are shown in Table 1. The PHQ-15 subscales and the total score were positively and significantly correlated with COVID-19 anxiety, as well as with GAD-7 total score. These correlations are in line with results from the first wave of the UK study [20]. Figure 1 shows the relationship between COVID-19 anxiety and somatic symptoms.

Table 2 shows the estimates from the regression models. In Model 1, considering COVID-19 first quintile as no-anxiety, we can observe significant differences between the baseline and the other quantiles both in PHQ-15 subscales and in its total score. In the sample, the aggravation of the somatic symptoms between quintiles seems to be characterized by a more linear increase than in the UK data, with a similar wider effect over the 5th quintile.

In Model 2, the control variables were taken into account. The effect of COVID-19 anxiety on somatic symptoms was weaker but similar to Model 1, especially referred to the total scale score. The presence of pre-existing health problems and high scores on GAD-7 significantly worsen specific and general somatic symptoms, confirming UK’s findings. Mourning for COVID-19 losses also has a detrimental effect on psychological health. No effect of household income was found. In terms of regions, Campania showed higher scores of somatic symptoms than the other regions. Campania scores were high in the PHQ-15 pain subscale and in the gastrointestinal subscale, but also overall.

### 3.2. Anxiety, Depression, and Traumatic Stress

We focused on anxiety, depression, traumatic stress and COVID-19 related anxiety, following Shevlin et al. (2020). In order to be consistent with the original study, traumatic stress and COVID-19 related anxiety where treated independently, whereas a new variable was computed to describe participants with anxiety, depression, or both. Sample size, percentages and adjusted odds ratio are summarized in Table 3, Table 4 and Table 5.

The rate of anxiety (GAD-7) in the overall sample was 21.59% (95% CI: 15.93%–20.72%), with no differences between regions (χ^2^_(3)_ = 4.07, *p* = 0.25) and with more women above the anxiety cut-off (11.75%, men: 6.45%; χ^2^_(1)_ = 15.94, *p* < 0.001). Depression (PHQ-9), with a rate of 21.4% (95% CI: 18.95%–24.03%), showed no differences between regions (χ^2^_(3)_ = 0.29, *p* = 0.96) but was significantly higher among women (12.52%), compared to men (8.86%; χ^2^_(1)_ = 5.82, *p* = 0.016).

The rate of anxiety and/or depression was 26.11 (95% CI: 23.48%–28.91%). No significant differences between regions were detected (χ^2^_(3)_ = 1.78 *p* = 0.61), whereas the gender difference was confirmed (women: 15.90%, men: 10.21%; χ^2^_(1)_ = 13.37, *p* < 0.001).

Using the ITQ scale, 25.92% of the sample was above the traumatic stress cut-off score (95% CI: 23.34%–28.77%). No difference was found between regions (χ^2^_(3)_ = 4.30, *p* = 0.23), while the gender effect was again significant (women: 15.11%, men: 10.88%; χ^2^_(1)_ = 7.16, *p* = 0.007). Finally, COVID-19 related anxiety was 18.30% overall (95% CI: 16.02%–20.82%). Regions showed a similar rate (χ^2^_(3)_ = 2.66, *p* = 0.44) whereas a gender difference was found (women: 12.14%, men: 6.17%; χ^2^_(1)_ = 20–65, *p* < 0.001).

**Figure 2:**
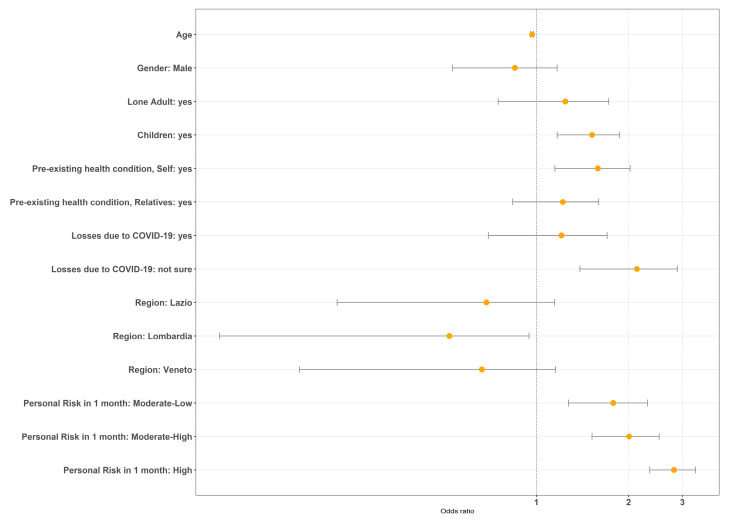
Plot of the odds ratio for the regression model of traumatic stress.

We considered anxiety and/or depression, traumatic stress, and COVID-19-related Anxiety as dependent variables in three separate multiple logistic regressions (respectively, Table 3, Table 4 and Table 5). Figure 2 shows the plot of the odds ratio for the model of traumatic stress.

The age effect, observed by Shevlin and colleagues, was confirmed for anxiety/depression (Adj OR = 0.96, CI = 0.95–0.97, *p* < 0.001), and traumatic stress (Adj OR = 0.97, CI = 0.96–0.98, *p* < 0.001), showing more moderate cases in younger participants. A gender difference was observed only in COVID-19 anxiety, with men less anxious than women about the new virus (Adj OR = 0.33, CI = −0.03–0.69, *p* < 0.001). No differences were observed between regions.

The presence of minors in the house had a detrimental effect on anxiety/depression (Adj OR = 1.55, CI = 1.20–1.90, *p* = 0.002) and traumatic stress (Adj OR = 1.52, CI = 1.17–1.87, *p* = 0.004), as did the presence of pre-existing health conditions in others (anxiety/depression; Adj OR = 1.53, CI = 1.14–1.91, p = 0.006) and themselves (traumatic stress; Adj OR = 1.59, CI = 1.15–2.02; *p* = 0.008).

Interestingly, an increase in perceived risk to contract COVID-19 in the following four weeks had an effect on depression/anxiety (LR χ^2^_(3)_ = 54.24, *p* < 0.001), traumatic stress (LR χ^2^_(3)_ = 66.14, *p* < 0.001), and COVID-19 anxiety (LR χ^2^_(3)_ = 84.74, *p* < 0.001), mostly in the third and fourth quartile.

## 4. Discussion

This report provides an overview of the Italian C19CRP study to investigate the psycho-social impact of the COVID-19 pandemic in the adult population. We aimed at replicating the analyses of the first two UK studies [19,20]. First, we focused on replicating the analyses by Shevlin et al. [20] about the somatic symptomatology and their relationship with anxiety. All the psychosomatic subscales showed positive correlations both with COVID-19 anxiety and general anxiety (GAD-7) (Table 1). In our regression analysis, higher COVID-19 anxiety was associated with more severe somatic symptomatology (Model 1, Table 2). Compared to the UK, in Italy the somatic symptoms showed a more linear relation between COVID-19 related anxiety and the somatic subscales and total score.

In Model 2, the individuals with higher COVID-19 anxiety displayed more severe somatic symptoms. When considering covariates, gender, health problems, GAD-7 ≥ 10, and human losses due to COVID-19 had a significant effect on somatic symptomatology. No consistent effect of region or household income were observed, with the exception of higher levels of somatic symptoms registered in the Campania region followed by the Veneto region.

In a second part of the analysis, we replicated the approach by Shevlin et al. [19]. We conducted three logistic regressions with different outcomes: presence of moderate anxiety/depression; presence of moderate traumatic stress symptoms; presence of high COVID-19-related anxiety.

When the outcome variable was the presence of moderate symptoms of anxiety and/or depression (Table 3), the factors associated with moderate symptoms of anxiety and/or depression were younger age, having children, having pre-existing precarious health conditions, mourning for COVID-19 losses, and perceiving a moderate to high risk of contracting the COVID-19 virus within one month. Regarding the regions, no significant results emerged.

If COVID-19 related anxiety is used as outcome (Table 4), being male had a protective effect, whilst living in a town and perceiving a low-moderate to high risk of getting COVID-19 within one month were associated with higher COVID-19 related anxiety.

When considering as dependent variable the presence of traumatic stress symptoms above the clinical cut-off (Table 5), younger age, having children, having pre-existing precarious health conditions, thinking about having lost someone due to COVID-19, and perceiving a low-moderate/high risk of contracting COVID-19 within one month were associated with more severe traumatic stress symptoms. Regarding, regional differences, living in Lombardia compared to Campania was associated with lower traumatic stress symptoms.

Across these models, an increase in the perceived risk to contract COVID-19 in the following month was associated with depression/anxiety, traumatic stress, and COVID-19 anxiety.

Summarizing, these results are in line with those of the UK study about anxiety, depression, and traumatic stress [19]. The UK and Italian data identify factors associated with worst psychological health: being a woman, younger age, having children, pre-existing health issues of self or someone close, and the moderate to high perceived risk of contracting COVID-19 within one month. Unlike Italy, in the UK lower household incomes and having (had) the COVID-19 were associated with worst psychological health outcomes.

Despite the similar variables used in Italy and in UK, the comparison has to take into account the differences in data collection in regard to the evolution of the epidemic. The first wave in the UK was launched at the beginning of the lockdown while the Italian first wave took place two months after the end of the first lockdown, thus focusing on the adaptation to life with COVID-19. The literature shows that psychological distress symptoms can last for years [13,33,34,35] and data from Italy confirms that the levels of psychological distress are still considerable two months after the end of the lockdown. Moreover, the literature about other infectious respiratory diseases (IRDs) (e.g., H1N1, MERS, SARS) shows that it is important to monitor psychological health also after the peak [34,35,36].

Regarding the differences among Italian regions, results suggest that in July 2020 the Campania and Lazio regions suffered a strong psychological impact of the COVID-19 pandemic. In particular in Campania somatic symptoms were more severe, compared to Lombardia and Veneto (Model 2). COVID-19-related anxiety was higher in Campania than in Lombardia (Table 3). This is counterintuitive given that Lombardia had been affected earlier and to a larger extent. There were no significant regional differences between regions in term of anxiety/depression and traumatic stress. As for COVID-19 related anxiety, this result appears counterintuitive, because on the basis of the differences in infection rate and media coverage one may have expected Lombardia to have higher scores than other regions.

There are a few factors that may have affected these results. As always, findings should be contextualized with respect to the time and circumstances of data collection. Lombardia and Veneto experienced COVID-19 issues for a longer period and had more contagions than Lazio and Campania. Resilience may have increased over time [37]. Another possible explanation concerns the role of media in transmission of threatening information, when the data was gathered people in areas with higher infection rates (e.g., north vs. south; Italy vs. UK) already received more threatening information, thus increasing their risk perception but also their strength. A second factor is that in July 2020 attention was focused on the central and southern regions because of seasonal tourism. This may be related to the higher anxiety and somatic issues in the Campania region at this point in time. Finally, as discussed in the introduction, one reason both Lombardia and Veneto were included in our study is because they are geographically contiguous but had been differentially affected by the pandemic. If differences reflect geography rather than extent of the health emergency, these may also relate to differences in attitudes or approach to the survey in different regions. Further investigations are necessary to clarify similarities and differences between regions.

These findings from the Italian general population suggest that two months after the end of lockdown the psychological distress was still present, and it was high for certain categories of the population, such as women, the younger, and those with children. Our results are in line with the current literature about the impact of COVID-19 in Italy [10,38,39,40] and worldwide [41] both in clinical and general population as well. To date, most of studies focused on the negative consequences of COVID-19. An Italian study in general population showed the COVID-19 massive effects with rates of psychological issues ranging around 41.8% for high distress, 32.1% for high anxiety, and 7.6% for PTSD symptomatology linked to the virus [38]. Other Italian studies focused on the mental health of specific categories, as parents [40] at higher risk of experiencing distress and young adults [39] who showed an increase in internalizing and externalizing problems, anxiety, depression, somatic complaints, and aggressive and rule-breaking behaviors.

The anxiety related to COVID-19 was associated with somatic symptomatology beyond general anxiety, thus suggesting that COVID-related issues (e.g., degree and type of exposure to COVID-19) deserve clinical consideration. Moreover, results highlighted the socio-demographic risk-factors associated with more severe psychological outcomes. These indicators are useful to identify at-risk groups in the population which may need targeted psychological interventions. Noteworthy, the perceived risk of contracting COVID-19 was considerable in the population. This may influence a number of outcomes ranging from psychological issues, hygiene practices, precautionary behavior, attitudes toward vaccine, trust in institutions, and beliefs in conspiracy theories. Future studies will focus on the role of other aspects that are relevant for adaptation to living with the COVID-19 in the Italian context.

The limitations of this study should be acknowledged. An online administration methodology was used, this may bias the results because there may be differences between the paper-pen administration and the online version. Self-report measures were used, with potential well-known biases related to social desirability and misleading answers. When the study was launched in UK, a broad number of measurement tools were used, including already validated measures and tools developed ad hoc in order to reflect the current psychological issues related to the COVID-19 emergency. Given that the same measures were administered in Italy, socio-cultural differences suggest caution in interpretation and in the direct comparison [20].

An observational cross-sectional study design was used, whilst a longitudinal design would be desirable to monitor the evolution of the COVID-19 impact. Future waves of the survey would provide valuable information. Our analyses can only show association and not causation. Finally, despite the well-balanced sample, a larger sample would better capture and represent minorities.

The Italian C19PRC can contribute to ongoing research on health issues such as: the factors hindering resilience, the relationship between conspiracy theories and denial, the link between trust in Institutions and compliance, the burden and clinical characteristics of psychological issues, and the psychological heath of specific subgroups at higher risk for adverse psychophysical outcomes. Moreover, comparing results in a broader international framework allows cross-cultural comparisons. In addition to Italy and the UK, the international consortium now includes the Republic of Ireland (from March 2020), Spain (from April 2020), the United Arab Emirates (from April 2020), and Saudi Arabia (from May 2020).

Regarding the strengths of this research, respondents were recruited by using an online research panel with stratified quota sampling to ensure that the sample would meet the characteristics of sex, age, household income of the Italian population. Moreover, the C19PRC Study was designed drawing from studies that investigated previous IRDs epidemics and their psychosocial impact (e.g., H1N1, MERS, SARS). To do so, several factors were assessed to capture the complexity of the phenomenon from an ecological perspective, taking into account the demographic, social, political, economic, and psychological influences. The findings of this project may be useful to understand and manage the psycho-social implications of the COVID-19 pandemic.

## 5. Conclusions

The social and psychological impact of the COVID-19 pandemic is believed to be severe and long-lasting. Behavioral changes and psychological impact need to be studied over time and across countries. Therefore, the Italy COVID-19 study was modelled on the UK COVID-19 study. This paper is only a first summary of the Italian data. Analyzing and combining large datasets will allow a better understanding of the phenomena and in turn the development of measure to safeguard the psychological health of individuals.

## Figures and Tables

**Table 1:** Correlation table for all the variables included in Model 1.

	Age	Gender	Income	C-19 Human Losses	P.H.I.	C-19 Anxiety	PHQ-15 (Pain)	PHQ-15 (Gastro)	PHQ-15 (Cardio)	PHQ-15 (Fatigue)	PHQ-15 (Total)
**Gender**	0.19 ***										
**Income**	0.00	0.12 ***									
**C-19** **Human Losses**	−0.12 ***	−0.09 **	−0.04								
**P.H.I.**	0.21 ***	0.09 **	−0.07 *	−0.05							
**GAD-7 (Total)**	−0.23 ****	−0.13 ****	−0.02	−0.28 ***	0.06						
**C-19 Anxiety**	−0.06	−0.14 ****	−0.05	0.31 ****	0.01	0.23 ****					
**PHQ-15 (Pain)**	0.07 *	−0.09 **	−0.08 *	0.15 ****	0.19 ****	0.25 ****	0.10 **				
**PHQ-15 (Gastro)**	−0.23 ****	−0.15 ****	0.00	0.25 ****	0.10 **	0.35 ****	0.10 **	0.46 ****			
**PHQ-15 (Cardio)**	−0.09 **	−0.13 ****	0.00	0.29 ****	0.19 ****	0.40 ****	0.12 ***	0.43 ****	0.59 ****		
**PHQ-15 (Fatigue)**	−0.07 *	−0.13 ****	−0.04	0.18 ****	0.18 ****	0.31 ****	0.13 ***	0.5***	0.56 ****	0.51 ****	
**PHQ-15 (Total)**	−0.08 *	−0.15 ****	−0.04	0.24 ****	0.20 ****	0.36 ****	0.12 ***	0.75 ****	0.82 ****	0.71 ****	0.81 ****

*Note*. * *p* < 0.05; ** *p* < 0.01; *** *p* < 0.001; **** *p* < 0.0001 (adjusted controlling the false discovery rate [32]). Abbreviations: C-19: COVID-19; P.H.I.: Previous Health Issues; GAD-7: Generalized Anxiety Disorder 7-item; PGQ-15: Patient Health Questionnaire-15.

**Table 2:** Regression coefficients from models predicting PHQ-15 scale and subscale scores.

	Pain	Gastro	Cardio	Fatigue	Total Score
**Model 1**					
COVID-19 anxiety					
Quintile 1	-	-	-	-	-
Quintile 2	0.18	0.61 **	0.33 *	0.23 *	1.36 **
Quintile 3	0.26 *	0.91 ***	0.55 ***	0.30 **	2.03 ***
Quintile 4	0.26 *	0.89 ***	0.53 ***	0.47 ***	2.15 ***
Quintile 5	0.56 ***	1.20 ***	0.90 ***	0.72 ***	3.40 ***
*R* ^2^	0.02 ***	0.04 ***	0.03 ***	0.04 ***	0.05 ***
**Model 2**					
COVID-19 anxiety					
Quintile 1	-	-	-	-	-
Quintile 2	0.13	0.42 *	0.22	0.14	0.92 *
Quintile 3	0.12	0.46 *	0.26	0.10	0.95 *
Quintile 4	0.08	0.37	0.19	0.23	0.87
Quintile 5	0.25 *	0.38	0.32	0.32 *	1.27 *
*Control variables*					
Age	0.006 **	−0.01 ***	0.00	0.00	−0.02
Gender (Male)	−0.18 *	−0.30 *	−0.24 *	−0.20 *	−0.93 **
Income (Max)	−0.12	0.08	0.10	0.00	0.04
Health Problem (Yes)	0.52 ***	0.63 ***	0.60 ***	0.50 ***	2.27 ***
GAD-7 ≥ 10	0.75 ***	1.82 ***	1.48 ***	0.87 ***	4.93 ***
Region					
Lazio	−0.19	−0.17	−0.12	−0.14	−0.63
Lombardia	−0.21 *	−0.15	−0.09	−0.19	−0.64
Veneto	−0.26 *	−0.38	−0.19	−0.18	−1.03 *
Human Losses due to COVID-19					
Yes	0.30 *	0.71 ***	0.37 *	0.41 ***	1.80 ***
Not sure	0.43 *	0.34	0.09	0.34*	1.21
*R* ^2^	0.14 ***	0.24 ***	0.22 ***	0.18 ***	0.27 ***

*Note*. * *p* <0.05; ** *p* <0.01; *** *p* <0.001 (adjusted controlling the false discovery rate [32]).

**Table 3:** Multiple logistic regression results predicting anxiety or depression.

	*n*	Anxiety/Depression (*n*%)	Adjusted OR (95% CI)
Age			
	1038	271 (26.10%)	0.962 (0.951–0.973) ***
Gender			
Female	531	165 (31.07%)	-
Male	507	106 (20.90%)	0.728 (0.406–1.048)
Living Location			
Rural	49	11 (22.45%)	-
Town	297	57 (19.19%)	0.960 (0.162–1.815)
Suburb	123	41 (33.33%)	1.648 (0.796–2.551)
City	569	162 (28.47%)	1.365 (0.592–2.200)
Lone Adult			
No	899	238 (26.47%)	-
Yes	139	33 (23.74%)	1.258 (0.759–1.744)
Children			
No	680	131 (19.26%)	-
Yes	358	140 (39.10%)	1.552 (1.203–1.902) **
Income			
−15.000 €/yr	218	66 (30.27%)	-
−28.000 €/yr	214	50 (23.36%)	0.709 (0.224–1.189)
−55.000 €/yr	212	50 (23.59%)	0.880 (0.380–1.377)
−75.000 €/yr	211	67 (31.75%)	1.039 (0.543–1.534)
+75.000 €/yr	183	38 (20.54%)	0.559 (0.009–1.100)
Pre-Existing Health Condition, Self			
No	869	222 (25.54%)	-
Yes	169	49 (29.00%)	1.356 (0.911–1.813)
Pre-Existing Health Condition, Relatives			
No	811	192 (23.67%)	-
Yes	227	79 (34.80%)	1.526 (1.145–1.906) **
Human Losses due to COVID-19			
No	888	217 (24.43%)	-
Yes	109	38 (34.86%)	1.519 (1.017–2.012) *
Not Sure	41	16 (39.02%)	2.027 (1.267–2.771) **
Region			
Campania	227	65 (28.63%)	-
Lazio	234	61 (26.06%)	0.972 (0.504–1.440)
Lombardia	391	94 (24.04%)	0.728 (0.292–1.165)
Veneto	186	51 (27.42%)	0.912 (0.407–1.414)
Personal Risk at 1 Month			
Low	269	33 (12.26%)	-
Moderate—Low	252	46 (18.25%)	1.435 (0.924–1.955)
Moderate—High	261	75 (28.73%)	1.930 (1.453–2.423) ***
High	256	117 (45.70%)	2.565 (2.104–3.047) ***

*Note*. * *p* < 0.05; ** *p* < 0.01; *** *p* < 0.001 (adjusted controlling the false discovery rate [32]).

**Table 4:** Multiple logistic regression results predicting COVID-19-related anxiety.

	n	C-19 Related Anxiety (n%)	Adjusted OR (95% CI)
Age			
	1038	190 (26.10%)	1.001 (0.989–1.014)
Gender			
Female	531	126 (23.73%)	-
Male	507	64 (12.62%)	0.336 (−0.028–693) ***
Living Location			
Rural	49	5 (10.20%)	-
Town	297	62 (20.87%)	2.111 (1.147–3.265) *
Suburb	123	26 (21.14%)	1.648 (0.796–2.551)
City	569	97 (17.04%)	2.035 (0.994–3.238)
Lone Adult			
No	899	170 (18.90%)	-
Yes	139	20 (14.38%)	0.629 (0.051–1.168)
Children			
No	680	112 (16.47%)	-
Yes	358	78 (21.79%)	1.001 (1.595–1.402)
Income			
−15.000 €/yr	218	51 (23.39%)	-
−28.000 €/yr	214	42 (19.62%)	0.721 (0.214–1.223)
−55.000 €/yr	212	28 (13.20%)	0.219 (−0.353–0.775)
−75.000 €/yr	211	33 (15.64%)	0.389 (−0.171–0.937)
+75.000 €/yr	183	36 (19.67%)	0.949 (0.386–1.507)
Pre-Existing Health Condition, Self			
No	869	157 (18.06%)	-
Yes	169	33 (19.52%)	1.015 (0.510–1.501)
Pre-Existing Health Condition, Relatives			
No	811	136 (16.77%)	-
Yes	227	54 (23.7%)	1.325 (0.904–1.738)
Human Losses due to COVID-19			
No	888	159 (17.90%)	-
Yes	109	23 (21.1%)	1.099 (0.541–1.628)
Not Sure	41	8 (19.51%)	1.283 (0.355–2.108)
Region			
Campania	227	49 (21.58%)	-
Lazio	234	42 (17.95%)	0.913 (0.402–1.421)
Lombardia	391	64 (16.37%)	0.719 (0.250–1.190)
Veneto	186	35 (18.81%)	0.935 (0.390–1.473)
Personal Risk at 1 Month			
Low	269	17 (6.32%)	-
Moderate–Low	252	33 (13.09%)	1.747 (1.134–2.392) *
Moderate–High	261	43 (16.47%)	1.956 (1.366–2.585) **
High	256	97 (37.89%)	3.196 (2.647–3.798) ***

***Note*.** * *p* < 0.05; ** *p* < 0.01; *** *p* < 0.001 (adjusted controlling the false discovery rate [32]).

**Table 5:** Multiple logistic regression results predicting traumatic stress.

	*n*	Traumatic Stress (*n*%)	Adjusted OR (95% CI)
Age			
	1038	269 (25.91%)	0.968 (0.957–0.979) ***
Gender			
Female	531	157 (29.56%)	-
Male	507	112 (22.09%)	0.850 (0.532–1.168)
Living Location			
Rural	49	13 (26.53%)	-
Town	297	59 (19.86%)	0.670 (−0.092–1.475)
Suburb	123	33 (26.83%)	0.940 (0.109–1.803)
City	569	164 (28.82%)	1.049 (0.312–1.832)
Lone Adult			
No	899	236 (26.25%)	-
Yes	139	33 (23.74%)	1.243 (0.749–1.722)
Children			
No	680	134 (19.70%)	-
Yes	358	135 (37.70%)	1.520 (1.169–1.87) **
Income			
−15.000 €/yr	218	60 (27.52%)	-
−28.000 €/yr	214	52 (24.30%)	0.906 (0.425–1.386)
−55.000 €/yr	212	58 (27.36%)	1.259 (0.771–1.749)
−75.000 €/yr	211	63 (29.85%)	1.099 (0.600–1.597)
+75.000 €/yr	183	36 (19.67%)	0.657 (0.102–1.203)
**Pre-Existing Health Condition, Self**			
No	869	216 (24.85%)	-
Yes	169	53 (31.36%)	1.587 (1.148–2.023) **
Pre-Existing Health Condition, Relatives			
No	811	197 (24.29%)	-
Yes	227	72 (31.71%)	1.219 (0.836–1.597)
Human Losses due to COVID-19			
No	888	219 (24.66%)	-
Yes	109	33 (30.27%)	1.207 (0.696–1.702)
Not Sure	41	17 (41.46%)	2.130 (1.386–2.886) **
Region			
Campania	227	70 (30.83%)	-
Lazio	234	57 (24.36%)	0.686 (0.223–1.146)
Lombardia	391	92 (23.53%)	0.519 (0.092–946) *
Veneto	186	50 (26.88%)	0.663 (0.168–1.154)
Personal Risk at 1 Month			
Low	269	29 (10.78%)	-
Moderate–Low	252	51 (20.24%)	1.782 (1.272–2.307) **
Moderate–High	261	69 (26.43%)	2.008 (1.519–2.518) ***
High	256	120 (46.87%)	2.815 (2.345–3.310) ***

*Note*. * *p* < 0.05; ** *p* < 0.01; *** *p* < 0.001 (adjusted controlling the false discovery rate [32]).

## Data Availability

Data is contained within the article or supplementary material. The data presented in this study are available in [Supplementary material S1 and Tables S1–S20].

## Supplementary Materials

The following are available online at https://www.mdpi.com/2077-0383/10/1/52/s1, Table S1: Age; Table S2: Ethnicity (frequencies); Table S3: Level of education (frequencies); Table S4: Occupation (frequencies); Table S5: Income (frequencies); Table S6: Financial concern (1–10); Table S7: Religious belief (frequencies); Table S8: Marital status (frequencies); Table S9: Individual and family health issues (frequencies); Table S10: COVID-19-related anxiety (0–100); Table S11: Perceived personal risk in the next 30 days (0–100); Table S12: COVID-19 total participants tested and confirmed cases (participants, relatives, friends); Table S13: Patient health questionnaire (PHQ-15, range: 0–30); Table S14: PHQ-15, pain symptoms (range 0–6); Table S15: Gastrointestinal symptoms (range 0–8); Table S16: PHQ-15, cardiopulmonary symptoms (range 0–8); Table S17: PHQ-15, fatigue symptoms (range 0–4); Table S18: Patient Health Questionnaire-9 (PHQ-9, range 0–27); Table S19: *International Trauma Questionnaire* (ITQ, range 0–36); Table S20: Generalized Anxiety Disorder 7-item Scale (GAD-7, range 0–21).
