# Supplementary material for: The Italian COVID-19 Psychological Research Consortium (IT C19PRC): General Overview and Replication of the UK Study
Source: J Clin Med. 2020 Dec 25;10(1):52. doi: 10.3390/jcm10010052 (PMC7796148; doi:10.3390/jcm10010052)
Supplement: Supplementary file 1 [file jcm-10-00052-s001.pdf]

Supplementary materials

The Italian Covid-19 Psychological Research Consortium (IT C19PRC): General Overview and Replication of the UK study

Giovanni Bruno[1], Anna Panzeri[1], Umberto Granziol[1], Fabio Alivernini[3], Andrea Chirico[3], Federica Galli[3], Fabio Lucidi[3], Andrea Spoto[1] and Giulio Vidotto[1], Marco Bertamini[1,2*]

[1] University of Padua, Italy;
[2] University of Liverpool, UK;
[3] University of Rome, Italy;
* Correspondence: m.bertamini@liv.ac.uk

Measures of the C19PRC Italian study – Adapted from McBride et al. 2020

Socio-demographic characteristics: Biological sex, Age, Ethnicity, Religious affiliation, Highest level of educational attainment, Gross annual household income, Current economic activity, Residential location (urban or rural), Household composition (number of adults and underaged children), Born in Italy and raised there (i.e., lived in Italy before the age of 16 years).

Health characteristics: Precarious health of self, Precarious health of beloved ones, Pregnancy (self and others) and week of gestation.

COVID-19:

Knowledge of COVID-19 symptoms. Respondents indicated which of 12 symptoms they believed to be the most commonly reported symptoms of COVID-19. Response format: Categories (yes/unsure/no)

Transmission of COVID-19. Respondents indicated which of 8 pathways of transmission of COVID-19 they believed were possible. Response format: Categories (yes/no)

Attitudes relating to risk of contracting COVID-19. Respondents rated their agreement with 6 statements about reducing the risk of contracting COVID-19 (e.g., washing hands with soap, using alcohol-based hand-rub; maintaining 1 meter between persons). Response format: a 5-point Likert scale ranging from 1 'completely disagree' to '5 completely agree'

Health Behaviours relating to preventing COVID-19 transmission. Participants rated the extent to which 17 statements based on the COM-B (Capability, Opportunity, Motivation-Behaviour, version 1) (Michie, Van Stralen, & West, 2011) were true for them during the COVID-19 pandemic. The health-protective behaviours were *maintaining hygienic practices* and *social distancing* to help prevent COVID-19 and reduce the spread of the disease. Response format: a 5-point Likert scale ranging from 1 'strongly agree' to 5 'strongly disagree'

Personal behaviour change to reduce individual risk of contracting COVID-19. Respondents reported whether and how they changed behaviour to reduce the risk of being infected by COVID-19. Response format: a 4-point Likert scale from 1 'Not at all' to 4 'Avoided completely'.

Experiences of COVID-19 symptoms, testing and diagnosis. Respondents reported if they experienced symptoms of COVID-19, whether they had been tested for the virus, the outcome (positive/negative) of that test, if someone close to them (e.g., a family member or friend) had experienced symptoms of COVID-19, if the outcome after

being tested for COVID-19 was positive or negative, and if they were currently (or had in the recent past) self-isolated. Response format: Categories (yes/unsure/no)

Anxiety relating to COVID-19. Respondents' rated on a visual analogue scale the specific anxiety about the COVID-19 pandemic. Response format: a visual slider scale ranging from 0 'not at all anxious' on the left to 100 'extremely anxious' on the right.

Perceived risk of contracting COVID-19. Respondents estimated the risk of contracting COVID-19 within 1 month, within 3 months, and within 6 months. They also estimated the perceived risk of different vulnerable groups (the elderly, children, pregnant women, and those with underlying chronic health conditions) experienced serious illness and (separately) death following a diagnosis of COVID-19. Response format: Visual sliders (ranging from 0% on the left-hand side to 100% on the right-hand side)

Knowledge of appropriate action to take following COVID-19 diagnosis. Respondents reported what members of the public had been advised to do if they suspected that they had been infected by COVID-19. Response format: Categories.

Attitudes relating to accepting any potential COVID-19 vaccine. Respondents expressed the acceptability of a new COVID-19 vaccine for themselves, their children, their relatives' children, and their elderly relatives. Response format: Categories (yes/no/maybe)

Consumer purchasing during COVID-19 pandemic. Respondents reported the extent to which they increased purchasing of various items (e.g., sanitary products, dried foods) in the weeks before the survey. Response format: a five-point scale ranging from (1) 'not at all' to 5 'very considerably'.

Perceived household income changes during the COVID-19 pandemic. 1) Respondents reported their experiences of (a) a loss of income due to not being able to work and about (b) financial savings due to changes in leisure activities. 2) Respondents reported their worries about how the pandemic affected their household finances. Response format: 1) Categories (yes/no/unsure); 2) Response options ranging from 1 'not at all worried' to 10 'extremely worried'.

Mental health

Depression was assessed with the *Patient Health Questionnaire-9 (PHQ-9)* (Kroenke, Spitzer, & Williams, 2001) a nine-item measure asking how often, over the last two weeks, each of the depressive symptoms was present. PHQ-9 scores range from 0 to 27, with scores of ≥5, ≥10, ≥15, representing mild, moderate and severe levels of depression severity (Kroenke et al., 2001). A threshold of ≥10 was used in this study. Response format: a 4 point Likert scale with 0 = 'not at all', 1='several days', 2='more than half the days', and 3='nearly every day'.

Generalized Anxiety Disorder Scale (GAD-7) (Spitzer, Kroenke, Williams, & Löwe, 2006) assessed the frequency of seven anxiety symptoms in the past 7 days. A cut-off score of 10 identifies generalised anxiety disorder (Spitzer et al., 2006). Response format: a 4-point Likert scale ranging from 1 (not at all) to 4 (nearly every day).

Persecution and Deservedness Scale (PaDS) (Melo, Corcoran, Shryane, & Bentall, 2009) assessed paranoia with five items (statements) from the persecution subscale of the persecution and deservedness scale (PaDS) (Elahi, Algorta, Varese, McIntyre, & Bentall, 2017). Response format: a 5-point scale ranging from 1 = strongly disagree to 5 = strongly agree.

International Trauma Questionnaire (ITQ) (Cloitre et al., 2018). The ITQ assessed Post-traumatic stress disorder in relation to the COVID-19 experience in the past month with 6 items across the three symptom clusters of Re-experiencing, Avoidance, and Sense of Threat. The functional impairment caused by these symptoms was

measured with three items. Response format: a 5-point Likert scale from 0 (Not at all) to 4 (Extremely) with possible PTSD scores ranging from 0 to 24.

Patient Health Questionnaire-15 (PHQ-15) (Kroenke, Spitzer, & Williams, 2002): it assesses the presence and severity of somatization disorder somatic symptoms experienced over the last week. Total score ranges from 0 to 30, scores of ≥5, ≥10, ≥15 represent mild, moderate and severe somatization levels. Response format: 0 ('not bothered at all'), 1 ('bothered a little') or 2 ('bothered a lot').

Treatment seeking behaviour for mental health difficulties. respondents reported their history of mental health treatment. Response format: Categories (never received; received in the past; currently receiving).

Psychological variables

Death Anxiety Inventory (DAI) (Tomás-Sábado, Gómez-Benito, & Limonero, 2005). It assessed the respondents' attitudes towards death by using 17-item across four death anxiety factors (death acceptance, externally generated death anxiety, death finality, thoughts about death). Response format: a 5-point Likert scale ranging from 1 'totally disagree' to 5 'totally agree'.

Big-Five Inventory (BFI-10) (Rammstedt & John, 2007). It assessed the five personality traits of openness to experience, conscientiousness, extroversion, agreeableness and neuroticism with two items per construct. Response format: a 5-point Likert type scale from 1 'totally disagree' to 5 'totally agree'.

Intolerance of uncertainty Scale (IUS) (Buhr & Dugas, 2002). It evaluates the intolerance of uncertainty that generates and maintain worry, with 12 items. Response format: a 5-point Likert scale ranging from 1 'not at all characteristic of me' to 5 'entirely characteristic of me'.

Loneliness Scale (Hughes, Waite, Hawkley, & Cacioppo, 2004). It measures social connectedness with 3 items about the frequency of loneliness. Response format: a 3-point scale (hardly ever, sometimes, or often).

Obsessive-Compulsive Inventory-Revised (OCI-R*)* (Marchetti, Chiri, Ghisi, & Sica, 2010)**.** With 18 items it measures the principal 6 dimensions characterizing the obsessive-compulsive disorder: *'Washing', 'Obsessing', 'Hoarding', 'Ordering', 'Checking', and 'Mental Neutralizing'.* Response format: a 5-point Likert type scale ranging from 0 (bothered not at all) to 4 (bothered a lot).

Single-Item Self-esteem Scale (SISES) (Robins, Hendin, & Trzesniewski, 2001). Respondents' reported their level of agreement with a single statement ('*I have high self-esteem'*). Response format: a 7-point Likert scale ranging from 1 'not very true of me' to 7 'very true of me'.

Brief Resilience Scale (BRS) (B. W. Smith et al., 2008). It assesses the level of trait resilience with 6 items, such as *'I tend to bounce back quickly after hard times'.* Response format: a 5-point Likert scale ranging from 1 'strongly disagree' to 5 'strongly agree'.

*Cognitive Reflection Task of Analytical Reasoning (CRT) (*Frederick, 2005) measures analytical reasoning ability with three problems and two additional ones. Each problem stimulates intuitive but incorrect answers whilst analytic reasoning. Response format: multiple choice with three foil answers (including the hinted incorrect answer).

Social and political attitudes and behaviours

Very Short Authoritarianism Scale (VSA) (Bizumic & Duckitt, 2018). The VSA was used to assess respondents' levels of right-wing authoritarianism (RWA) and includes 6 items such as: *'It's great that many young people today are prepared to defy authority*'; '*What our country needs most is discipline, with everyone following our leaders in unity*'; and '*Our society does NOT need tougher government and stricter laws'*. Response format: a 5-point Likert scale ranging from 1 'strongly disagree' to 5 'strongly agree'.

Social Dominance Scale (SDO_7) (Ho et al., 2015). Social dominance orientation levels were assessed using the SDO7 with 8 items such as *'Some groups of people are simply inferior to other groups'.* Response format: a 5-point Likert scale ranging from 1 'Strongly oppose' to 5 'Strongly Favour'.

Identification with all humanity scale (IWAH) (McFarland, Webb, & Brown, 2012). The nine items were adapted for use in this study with reference to the 'Italians'. Respondents reported how much they felt identified with people in their community; people from Italy; and all humans everywhere. Response format: from 1 'not at all' to 5 'very much'.

Patriotism/Nationalism. Patriotism (three items) was referred to as 'constructive nationalism' has been conceptualised as love for one's country, whereas nationalism (two items) has been conceptualised as the belief that one's country is superior to others. Items to measure patriotism and nationalism were adapted from Davidov (2011). Response format: a 5-point Likert scales from 1 'strongly disagree' to 5 'strongly agree'.

Attitude towards migrants. It assessed attitudes towards migrants with 3 items adapted from the British Social Attitudes Survey 2015 (British Social Attitudes Survey 2015, 2015): (1) '*Would you say it is generally bad or good for Italy's economy that migrants come to Italy from other countries*?' (2) '*Would you say that Italy's cultural life is generally undermined or enriched by migrants coming to live here from other countries?*' and (3) '*Some migrants make use of Italy's schools, increasing the demand on them. However, many migrants also pay taxes which support schools and some also work in schools. Do you think that, on balance, migration to Italy reduces or increases pressure on the schools*?'. Response format: 1) scored on a 10-point scale ranging from 1 'extremely bad' to 10 'extremely good'; 2) scored on a 10-point scale ranging from 1 'undermined' to 10 'enriched'; 3) scored on a 5-point scale ranging from 1 'reduces pressure a lot' to 5 'increases pressure a lot'?

Voting behaviour and political party affiliation. Respondents reported: (1) if they had voted the last time; 2) which political party they voted for; (3) their political affiliation, (4) their views on social issues such as abortion and same-sex marriage and (5) their views on economic issues such as taxes and government spending. Response format: (1) categorial (yes/no); (2) categorial (political parties); (3) a 10-point scale ranging from 1 'left-wing' to 10 'right-wing'; (4) and (5) a 10-point scale from 1 'very liberal' to 10 'very conservative'.

Conspiracy mentality scale (CMS) (Imhoff & Bruder, 2014). It evaluates a generalized political attitude with five items such as: *'I think that there are secret organizations that greatly influence political decisions'.* Response format: a 5-point scale from 1 'Certainly not 0%' to 11 'Certainly 100%'

Trust in institutions. Respondents rated the extent to which they have trust in the institutions/groups such as political parties, government, scientists and health professionals. Response format: a 5-point Likert scale ranging from 1 'completely trust' to 5 'do not trust at all'.

Belongingness in neighbourhood. It assessed the level of belongingness and connectedness to the neighbourhood with three questions taken from the UK Community Liver Survey (Cabinet Office, 2015): *(1) 'How strongly do you feel you belong to your immediate neighbourhood?'; (2) 'How comfortable would you be with asking a neighbour to keep a set of keys to your home for emergencies?'; (3) 'How comfortable would you be asking a neighbour to collect a few shopping essentials for you, if you were ill and at home on your own?'.* Response format: 1) a 4-point scale from 1 'not at all' to 4 'very strongly'; 2) a 4-point scale ranging from 1 'very uncomfortable' to 4 'very comfortable'; 3) a 4-point scale ranging from 1 'very uncomfortable' to 4 'very comfortable'.

Religious identity and belief. Participants selected their religious identity among 'atheist', 'agnostic' and 'other'. Also, the Monotheist and Atheist Beliefs Scale (Alsuhibani, Shevlin, & Bentall, 2020, as cited in McBride et al., 2020) has 8 statements measuring religiosity (4 items) vs atheism (4 items). Response format: 5-point Likert scale, from 1 (strongly agree) to 5 (strongly disagree).

Table S1: Age

	N		min	1st Quartile	Mean	Median	3rd Quartile	max	Std Deviation
Overall	1038		18	34	48.2	49	62	87	16.14
Gender									
F	531		18	34	47.02	47	60	81	15.36
M	507		18	41	53	55	67	87	16.39
Region									
Campania	227		18	34	48.2	49	62	78	16.01
Lazio	234		20	40.25	51.77	54	64	87	15.46
Lombardia	391		18	36	50.76	51	66	86	16.33
Veneto	186		20	34	48.04	50	61	83	16.47

Table S2: ethnicity (frequencies)

	N		Asiatic	American	Arab	European	Indian	Others
Overall	1038		3	3	1	775	8	248
Gender								
F	531		3	2	1	390	3	3
M	507		0	1	0	385	5	0
Region								
Campania	227		0	1	0	155	3	68
Lazio	234		1	1	1	175	2	54
Lombardia	391		1	1	0	306	2	81
Veneto	186		1	0	0	139	1	45

Table S3: Level of Education (frequencies)

	N		None	Elementary	Secondary	High School	Bachelor	Master of Science	PhD	Professional Qualification
Overall	1038		0	3	83	506	97	297	28	24
Gender										
F	531		0	0	43	261	48	151	18	10
M	507		0	3	40	245	49	146	10	14
Region										
Campania	227		0	0	13	127	17	62	5	3
Lazio	234		0	1	16	111	17	77	7	5
Lombardia	391		0	1	35	176	39	114	11	15
Veneto	186		0	1	19	92	24	44	5	1

Table S4: Occupation (frequencies)

	N		Full Time	Partial Time	Unemployed (searching)	Unemployed	Retired	Student	Invalid
Overall	1038		461	99	112	53	251	57	5
Gender									
F	531		218	62	78	45	87	38	3
M	507		243	37	34	8	164	19	2
Region									
Campania	227		89	18	40	9	47	23	62
Lazio	234		105	24	31	13	51	8	77
Lombardia	391		189	34	18	19	114	16	114
Veneto	186		78	23	23	12	39	10	44

Table S5: Income (frequencies)

	N		– 15.000 €/year	- 28.000 €/year	- 55.000 €/year	- 75.000 €/year	+ 75.000 €/year
Overall	1038		218	214	212	211	183
Gender							
F	531		131	118	104	100	78
M	507		87	96	108	111	105
Region							
Campania	227		72	59	39	23	34
Lazio	234		56	36	44	57	41
Lombardia	391		55	74	91	91	80
Veneto	186		35	45	38	40	28

Table S6: Financial Concern (1-10)

	N		min	1st Quartile	Mean	Median	3rd Quartile	max	Std Deviation
Overall	1038		1	4	6.15	7	8	10	2.77
Gender									
F	531		1	4.5	6.44	7	8	10	2.76
M	507		1	4	5.85	6	8	10	2.77
Region									
Campania	227		1	5	6.42	7	8	10	2.65
Lazio	234		1	4	6.28	7	8	10	2.88
Lombardia	391		1	3	5.90	6	8	10	2.80
Veneto	186		1	4	6.20	7	8	10	2.71

Table S7: Religious Belief (frequencies)

	N		Christian	Muslim	Jewish	Buddhist	Atheist	Agnostic	Others
Overall	1038		772	2	4	6	152	69	33
Gender									
F	531		406	2	3	3	73	29	15
M	507		366	0	1	3	79	40	18
Region									
Campania	227		171	1	0	0	32	17	6
Lazio	234		163	0	1	1	45	15	9
Lombardia	391		295	0	3	3	52	24	14
Veneto	186		143	1	0	2	23	13	4

Table S8: Marital Status (frequencies)

	N		Never Married	Separated	Divorced	Widowed	Married	Others
Overall	1038		276	44	53	32	602	31
Gender								
F	531		406	2	3	3	73	29
M	507		366	0	1	3	79	40
Region								
Campania	227		74	12	6	7	126	2
Lazio	234		52	14	16	8	134	10
Lombardia	391		92	12	20	13	241	13
Veneto	186		58	6	11	4	101	6

Table S9: Individual and Family Health issues (frequencies)

	N		Health Issues - Personal		Health Issues - Family	
			Yes	No	Yes	No
Overall	1038		169	869	227	811
Gender						
F	531		70	461	125	406
M	507		99	408	102	405
Region						
Campania	227		36	191	64	163
Lazio	234		42	192	53	181
Lombardia	391		58	333	71	320
Veneto	186		33	153	39	147

Table S10: Covid-19 Related Anxiety (0-100)

	N		min	1st Quartile	Mean	Median	3rd Quartile	max	Std Deviation
Overall	1038		0	30	54.21	60	76	100	28.04
Gender									
F	531		0	44	59.99	63	80	100	26.39
M	507		0	20	48.15	51	71	100	28.47
Region									
Campania	227		0	42	57.95	61	79.50	100	27.09
Lazio	234		0	30	53.96	59.50	75	100	28.88
Lombardia	391		0	30	52.79	60	76	100	28.00
Veneto	186		0	30	52.93	51	75	100	28.01

Table S11: Perceived Personal Risk in the next 30 days (0-100)

	N		min	1st Quartile	Mean	Median	3rd Quartile	max	Std Deviation
Overall	1038		0	10	35.28	34	51	100	25.50
Gender									
F	531		0	12.50	37.57	40	53	100	26.06
M	507		0	10	32.88	30	51	100	24.70
Region									
Campania	227		0	10	35.47	39	51.50	100	25.54
Lazio	234		0	10.25	33.37	30	51	100	24.24
Lombardia	391		0	10	35.24	32	52.50	100	26.10
Veneto	186		0	17	37.53	35.50	51	100	25.73

Table S12: Covid-19 total participants tested & confirmed cases (participants, relatives, friends)

	N		Personal, Total Tested	Cases		
				Personal, confirmed	Relatives, confirmed	Friends, confirmed
Overall	1038		141	14	26	185
Gender						
F	531		74	7	10	98
M	507		67	7	16	87
Region						
Campania	227		22	1	6	31
Lazio	234		29	2	3	28
Lombardia	391		66	7	14	97
Veneto	186		24	4	3	29

Table S13: Patient Health questionnaire (PHQ-15, range: 0-30)

	N	Medium Severity (10-14)	High Severity (15-30)		min	1st Quartile	Mean	Median	3rd Quartile	max	Std Deviation
Overall	1038	95	43		0	1	4.13	3	6	26	4.78
Gender											
F	531	63	31		0	1	4.85	3	7	26	5.20
M	507	32	12		0	0	3.39	2	5	26	4.18
Region											
Campania	227	24	14		0	1	4.79	3	6	26	5.15
Lazio	234	17	6		0	1	3.84	3	5.75	26	4.32
Lombardia	391	36	17		0	1	4.07	2	6	26	4.94
Veneto	186	18	6		0	0.25	3.83	2	6	26	4.48

Table S14: PHQ-15, Pain Symptoms (range 0-6)

	N		min	1st Quartile	Mean	Median	3rd Quartile	max	Std Deviation
Overall	1038		0	0	1.39	1	2	6	1.47
Gender									
F	531		0	0	1.60	1	3	6	1.56
M	507		0	0	1.17	1	2	6	1.34
Region									
Campania	227		0	0	1.61	1	3	6	1.54
Lazio	234		0	0	1.32	1	2	6	1.43
Lombardia	391		0	0	1.35	1	2	6	1.47
Veneto	186		0	0	1.30	1	2	6	1.42

Table S15: PHQ-15, Gastrointestinal Symptoms (range 0-8)

	N		min	1st Quartile	Mean	Median	3rd Quartile	max	Std Deviation
Overall	1038		0	0	0.95	0	1	8	1.62
Gender									
F	531		0	0	1.13	0	2	8	1.78
M	507		0	0	0.77	0	1	8	1.41
Region									
Campania	227		0	0	1.15	0	2	8	1.80
Lazio	234		0	0	0.87	0	1	8	1.45
Lombardia	391		0	0	0.97	0	1	8	1.64
Veneto	186		0	0	0.81	0	1	8	1.54

Table S16: PHQ-15, Cardiopulmonary Symptoms (range 0–8)

	N		min	1st Quartile	Mean	Median	3rd Quartile	max	Std Deviation
Overall	1038		0	0	0.74	0	1	8	1.50
Gender									
F	531		0	0	0.93	0	1	8	1.69
M	507		0	0	0.55	0	0	8	1.26
Region									
Campania	227		0	0	0.87	0	1	8	1.62
Lazio	234		0	0	0.66	0	1	8	1.38
Lombardia	391		0	0	0.75	0	1	8	1.57
Veneto	186		0	0	0.70	0	1	8	1.37

Table S17: PHQ-15, Fatigue Symptoms (range 0-4)

	N		min	1st Quartile	Mean	Median	3rd Quartile	max	Std Deviation
Overall	1038		0	0	1.04	1	2	4	1.14
Gender									
F	531		0	0	1.18	1	2	4	1.20
M	507		0	0	0.89	1	2	4	1.06
Region									
Campania	227		0	0	1.19	1	2	4	1.18
Lazio	234		0	0	0.99	1	2	4	1.11
Lombardia	391		0	0	0.99	1	2	4	1.13
Veneto	186		0	0	1.01	1	2	4	1.15

Table S18: Patient Health Questionnaire-9 (PHQ-9, range 0-27)

	N	Above Cut-off (10)		min	1st Quartile	Mean	Median	3rd Quartile	max	Std Deviation
Overall	1038	222		0	2	6.69	5	9	27	5.85
Gender										
F	531	130		0	3	7.44	6	10	27	6.05
M	507	92		0	1.5	5.90	5	9	27	5.52
Region										
Campania	227	48		0	3	7.15	6	10	27	5.77
Lazio	234	51		0	2	6.66	5	9	27	5.96
Lombardia	391	81		0	2	6.29	5	9	27	5.84
Veneto	186	42		0	2	7	6	10	27	5.80

Table S19: *International Trauma Questionnaire* (ITQ, range 0-36)

	N	Above Cut-off (10)		min	1st Quartile	Mean	Median	3rd Quartile	max	Std Deviation
Overall	1038	269		0	1	9.49	7	17	36	8.76
Gender										
F	531	157		0	2	10.57	9	18	36	8.96
M	507	112		0	1	8.37	6	14	36	8.41
Region										
Campania	227	70		0	2	10.01	7	18	35	8.90
Lazio	234	57		0	1	9.59	8	17	36	8.56
Lombardia	391	92		0	1	9.32	7.5	16	36	8.73
Veneto	186	50		0	1	9.11	6	16	36	8.93

Table S20: Generalized Anxiety Disorder 7-item Scale (GAD-7, range 0-21)

	N	Above Cut-off (10)		min	1st Quartile	Mean	Median	3rd Quartile	max	Std Deviation
Overall	1038	189		0	1.25	5.74	5	8	21	5.15
Gender										
F	531	122		0	2	6.58	6	10	21	5.32
M	507	67		0	1	4.86	4	7	21	4.81
Region										
Campania	227	49		0	2	6.38	6	9	21	5.13
Lazio	234	36		0	1	5.49	5	7	21	4.87
Lombardia	391	66		0	1	5.29	4	8	21	5.16
Veneto	186	38		0	2	6.22	5	9	21	5.39
